# The involvement of *TNFRSF25* in age-related hearing loss

**DOI:** 10.1007/s00439-026-02826-5

**Published:** 2026-03-26

**Authors:** Marie Valerie Roche, Pei-Ciao Tang, Denise Yan, Michelle Rose De Marchena, Maria Camila Robayo, Clemer Abad, Yan Guo, Feng Gong, Katherina Walz, Xue Zhong Liu

**Affiliations:** 1 Department of Otolaryngology, University of Miami Miller School of Medicine, 1120 NW 14th Street, 5th Floor, Miami, FL 33136 USA; 2 Department of Biochemistry and Molecular Biology, University of Miami Miller School of Medicine, Miami, FL 33136 US; 3 John P. Hussman Institute for Human Genomics; John T Macdonald Foundation Department of Human Genetics, University of Miami Miller School of Medicine, Miami, FL 33136 USA; 4 Department of Public Health and Sciences, University of Miami Miller School of Medicine, Miami, FL 33136 US; 5 Departamento de Ingenieria Biomedica, Universidad Austral, CONICET, Pilar, Argentina; 6 Interdisciplinary Stem Cell Institute, University of Miami Miller School of Medicine, Miami, FL USA

## Abstract

Hearing loss is a prevalent sensory condition that affects the ability to perceive sounds. Hair cells play a vital role in hearing by converting mechanical sound vibrations into electrical signals that are transmitted to the brain. In humans, if damaged, these specialized sensory cells do not regenerate, leading to reduced sensitivity to sound, difficulty understanding speech, and deafness. The incidence of hearing loss increases with age, but it can also occur earlier due to genetic predisposition or environmental factors such as exposure to noise or ototoxic medications. Epigenetic mechanisms play a significant role in age-related hearing loss (ARHL) by regulating gene expression without changing the underlying DNA sequence. Aberrant DNA methylation patterns have been linked to ARHL. In this study, by performing an epigenome-wide association study in a cohort of 30 ARHL patients, we found a correlation between a specific CpG site methylation level proximal to the *TNFRSF25* gene, with an impaired hearing threshold. Interestingly, *Tnfrsf25* absence in mice cause ARHL, severe hair cell depletion, and degeneration of nerve fibers. Taking together these results suggests a crucial role of *Tnfrsf25* expression in hearing maintenance.

**Supplementary Information:**

The online version contains supplementary material available at 10.1007/s00439-026-02826-5.

## Introduction

Age-related hearing loss (ARHL), also called presbycusis, is a common form of sensorineural hearing loss affecting the elderly population. As the human life expectancy increases (Vollset et al. 2024), number of people who is affected by the ARHL is expected to increase. A recent study showed that more than 60% of those aged 71 or older has certain degree of hearing loss in US and by the age of 90 years old, more than 95% of adults has the hearing impairment (Reed et al. 2023). Though many factors have been suggested to contribute to ARHL, including the degeneration of hair cells and sensory neurons, exposure to the noise, underlying medical conditions, and genetics, the exact mechanism underlying ARHL remains elusive (Cunningham and Tucci 2017).

Epigenetic events are changes that can alter gene expression levels without changing the genomic DNA sequence. Mounting evidence indicates the crucial role of epigenetic mechanisms in hearing, by creating unique chromatin states and regulating gene expression in a cell type specific manner, which is vital for the inner ear’s development, long-term maintenance, and normal function. Epigenetic regulators include DNA methylation, histone modifications, ATP-dependent chromatin remodeling, and non-coding RNAs. They regulate the assembly of chromatin, thereby regulating gene expression and modifying genetic material accessibility in both the inner ear development and disease (Doetzlhofer and Avraham 2017, Mittal et al. 2020).

DNA methylation is the addition of a methyl groups to cytosine at the 5” position to CpG dinucleotides via the usage of three enzymes, DNMT1, DNMT3A and DNMT3B. They each participate in mammalian development (Okano et al. 1999). DNMT1 have been implicated in the inner ear and the maintenance of gene methylation in both central and peripheral neurodegeneration in hereditary sensory neuropathy with dementia (Klein et al. 2011). DNTMT3A/B work as *de novo* methyltransferase by establishing new methylation patterns, removal of these enzymes leads to mice lethality stemming from decrease genomic methylation (Li et al. 1992, Okano et al. 1998). The frequency of CpG methylation in the human genome are divided into two fractions with respective properties: (1) about 98% CpG are infrequent and methylated and (2) the remaining unmethylated sites are concentrated withing CpG islands, which colocalize with transcription start sites (TSSs) of genes (Bird 2002). The presence or absence of DNA methylation determines whether the status of TSS is active or silent (Illingworth et al. 2008). It was shown that CpG site methylation plays a role in the development and function of the inner ear within the cochlea sensory epithelium (Mittal et al. 2020). Furthermore, recent studies have related levels of DNA methylation to hearing loss (Patil et al. 2024) and ARHL (Kuo et al. 2021, Roche et al. 2025).

In this study, an epigenome-wide association study was conducted in a cohort of ARHL patients (*N* = 30). Our results showed a correlation between a specific CpG site methylation level proximal to the tumor necrosis factor receptor superfamily, *TNFRS25* gene (OMIM: 603366), with impaired hearing threshold. To our knowledge, TNFRSF25, a pro-inflammatory cytokine also known as death receptor 3 (DR3), was not previously related to ARHL. By generating and characterizing a *Tnfrsf25* knockout (*Tnfrsf25*^*−/−*^) mouse, we were able to correlate the absence of *Tnfrsf25* in mice to ARHL. Moreover, severe hair cell depletion and degeneration of nerve fibers were observed in the cochlea of *Tnfrsf25* knockout (*Tnfrsf25*^*−/−*^) mice in an age-dependent manner. Taking together these results suggests a crucial role of *Tnfrsf25* expression in hearing maintenance.

## Materials and methods

### Subjects

This study was approved by the University of Miami institutional review board. Subjects were recruited from adults attending the outpatient clinic the University of Miami Ear Institute. The study was performed in accordance with relevant guidelines and regulations. Written informed consent was obtained from all subjects prior to the clinical evaluation and blood sample collection. Genomic DNA was isolated from the blood sample and the DNA specimen was stored at −20 °C until the further analysis. A major hurdle in hearing loss is the lack of biopsy tissues from patients that can be studied at the molecular, cellular and pathological levels. Using DNAs and cell lines from patients with HL and animal models is the first step for genetic and epigenetic investigations in human auditory disorders (Mittal et al. 2020, Roche et al. 2025).

All patients completed a questionnaire on demographic information including ethnicity and a medical history focusing on the identification of factors with known effects on hearing such as excess noise, ear diseases, ear trauma, radiation exposure of surgery, chronic illness (i.e., diabetes, cardiovascular disease), manifestations of syndromic deafness (i.e., blindness from retinitis pigmentosa, tegumentary and craniofacial anomalies), and family history of hearing loss. Simplex and multiplex families were recruited.

The inclusion criteria for 30 subjects with ARHL consisted of age greater than 50 years with bone conduction pure tone average ([PTA]) of frequencies 5, 10, 20, and 40 kHz greater than 30 dB. The average age of the subjects was 63 ± 10 years. Exclusion criteria included (1) previous history of exposure to excess environmental and occupational noise; (2) exposure to toxins or drugs with known ototoxic effects; (3) history of temporal bone trauma; (4) history of otologic disorders such as Meniere’s disease, autoimmune hearing loss; (5) average conductive hearing loss greater than 15 dB hearing loss in one or two ears, measured at 5,10, and 20 kHz; (6) unilateral or significantly symmetric (greater than 25 dB difference in interaural PTA) hearing loss (Angeli et al. 2012, Bared et al. 2010).

### Data analysis

The Illumina MethylationEpic BeadChip microarray was used to perform an epigenome-wide association study. The 850 K methylation array data underwent processing, quality control, and normalization procedures using Illumina GenomeStudio software. Samples that failed quality control were removed from subsequent analysis. Human genome (hg38) was used as a reference. The relationship between hearing loss and CpG methylation levels was assessed through regression analysis using glm package in R. Hearing loss status served as the outcome variable, and each methylation level of each CpG site served as the primary predictor adjusting for age, sex, and race. Hearing loss was represented both as a binary (logistic regression) and continuous (linear regression) variable across hearing tests at different frequencies. Benjamini Hochberg multiple test corrected *p* < 0.05 was used as the significant threshold (Ferreira 2007). CpG sites were annotated to genes based on the Genecode v44 GTF, with annotations extending 1500 bp both upstream and downstream of the genes.

### Generation of *Tnfrsf25* knock-out mouse

To study the role of *Tnfrsf25* gene in hearing, a *Tnfrsf25* knockout mouse model (C57BL/6J) was generated by CRISPR/Cas-mediated genome engineering. The *Tnfrsf25* gene (NCBI Reference Sequence: NM_001291010.1; Ensembl: ENSMUSG00000024793) is located on mouse chromosome 4, and ten exons were identified, with the ATG start codon in exon 1 and the TGA stop codon in exon 10 (Transcript Tnfrsf25-201: ENSMUST00000025706). Exons 1 and 10 were selected as target sites. Four gRNAs (Table S1) were designed to delete a ~ 4186 bp region spanning exons 1 to 10 of *Tnfrsf25*.

*Tnfrsf*25 knockout homozygous founder animals C57BL/6J-Tnfrsf25^em1C^/Cya (from now on referred to as *Tnfrsf25*^*−/−*^), were generated by Cyagen. Founder mice were delivered to the University of Miami and used for colony expansion by breeding with wild-type C57BL6/J mice. All generated mice were weaned at 21 days old. 1–2 mm tail clip will be used for DNA isolation using QuickExtract (Biosearch Technologies) followed by PCR for genotyping using the PCR primers *Tnfrsf25* F1: 5’-GAAGATAAAGGCCAACATCCCAA-3’, *Tnfrsf25* R1: 5’- ATTGTACCTGCTCCTCATCCTATC-3’, and *Tnfrsf25* R2: 5’- TAGGTCGCCGGTGAAGACTAC-3’. PCR was carried out in standard conditions with an annealing temperature of 60℃ for 35 cycles. The amplification of a 446 bp product with primers F1 and R1 is expected for the deleted allele. To examine the unaltered gene, an amplification of a 742 bp is expected with primers F1 and R2. At weaning age, mice were housed in groups of two to five per cage with access to food and water. The animals were kept at room temperature 22 ± 2 °C, relative humidity 55 ± 10% and a light cycle of 12: 12 H (6: 00 a.m. to 6:00 p.m.). All procedures were approved by the University of Miami Institutional Animal Care and followed by the National Institute of Health (NIH) Guidelines, ‘Using Animals in Intramural Research’.

### Expression of the *Tnfrsf25* gene

Various tissues, including the cochlea, were isolated from P0 WT mice and transferred to 250 µl TRIzol (Invitrogen, Cat.No.15596026). The tissues were lysed and completely homogenized by ultrasound. 200 µl of chloroform was added to the TRIzol-Lysate mixture. The samples were incubated at room temperature for 5 min, then centrifuged at maximum speed for 20 min at 4 °C. The supernatant was transferred to a RNeasy Mini spin column placed in a 2 ml collection and processed following the manufacturer’s protocols (Qiagen.Cat.No.74104). The RNA yielded was quantified using a nanodrop spectrophotometer (ND-100, Thermo Fisher).

The isolated RNA was reverse transcribed to cDNA using the qScript™ XLT cDNA Supermix kit following the manufacturers’ protocols (QuantBio, Cat.No.95151-500). The volume of RNA added into each reaction was adjusted to a final concentration of 100 ng/µl. *Tnfrsf25* gene expression was evaluated by quantitative Realtime-PCR (qRT-PCR) using Taqman assays (ThermoFisher). *Gapdh* amplification was used as a reference control gene.

### Auditory brainstem recording (ABR)

The auditory brainstem response (ABRs) was used to assess the role of *Tnfrsf25* in the hearing capacity using a Smart EP Universal Smart Box (Intelligent Hearing Systems, Miami FL, United States. Each animal was anesthetized with an intraperitoneal injection of a mix of ketamine/xylazine (120/10 mg/kg body weight) before testing. A heating pad was used to maintain the body temperature of the tested mice, and during the recovery period at 37 °C. ABR stimuli were of 0.1 ms duration, clicks pure tone pips presented with frequencies 8, 16 and 24 kHz. Stimuli started at 20 dB SPL amplitude and increased at 10 dB steps up to 100 dB SPL, averaging a total of 600 sweeps for each frequency and amplitude. The ABR thresholds were defined by identifying the lowest stimulus level producing an identifiable ABR pattern; at minimum of two consistent peaks above the baseline. ABR was recorded in both WT and *Tnfrsf25* mutant animals (*N* ≥ 5).

#### Immunostaining of the cochlea

Cochlea specimens were collected WT and *Tnfrsf25*^−/−^ mice at postnatal day 0 (P0) followed by fixation in PBS containing 4% (vol/vol) paraformaldehyde (PFA) at 4 °C with agitation overnight. 45 μm sections were obtained with a Leica VT 1200 S vibratome. For the whole mount tissue, whole cochleae were dissected from 2 month-, 4 month- and 8 month-old WT and *Tnrfrs25*^−/−^ mice cochleae and fixed in 4% PFA/PBS overnight at 4 °C. The middle turns of the cochlea and the organ of Corti within the cochlea were dissected.

All specimens weree treated with a blocking buffer (PBS with 5% BSA and 1% Tritton X-100) for 1.5 h at room temperature. The samples were immunostained with the following primary antibodies : (1) DR3/Tnfrsf25 goat anti-mouse (R&D System, Minneapolis, MN, Cat.NoAF2437) for Tnfrsf25 visualization, (2) An hair cell specific marker : Mouse monoclonal anti-Myo7a (1/400 Developmental Studies Hybridoma Bank at the University of Iowa Cat.No.138-1), (3) Chicken polyclonal anti-neurofilament (NF) (1/300 Millipore, Cat.No.AB5539) was used for neuronal fibers visualization. The following secondary antibodies were used: (1) Anti-rabbit Alexa Fluor 488 (1/400, Invitrogen, Cat.No.A32731), (2) Anti-chicken, Fluor 488 (1/300, Invitrogen, Cat.No.A323931), (3) Anti-mouse Alexa Fluor 488 (1/400, Invitrogen, Cat.NoA32723), (4) Anti-rabbit Alexa Fluor 568 (1/400, Invitrogen Cat.No.A-11008), (5) Anti-mouse Alexa Fluor 568 (1/400, Invitrogen, Cat.No.A-21124). Images were obtained using a 63X objective with a Leica SP5 Inverted Confocal or a Zeiss LSM980 confocal microscope.

### Cell counting

To quantify cell density, in each sample 4 independent regions of identical size were selected from the images. Outer hair cells and Inner hair cells were counted manually based on the staining of Myo7a and DAPI.

### Statistical analysis

For the human studies, statistical analysis including linear and logistic regressions, were conducted using R v 4.3.0. p-value < 0.05 was considered statistically significant. Multiple test corrections were conducted when necessary, using the false discovery rate method. Correlation tests were computed using Pearson’s correlation. Principal component analysis (PCA) was performed using Python. Heatmap and unsupervised clustering analysis was carried out using the heatmap package in R (Subramanian et al. 2005, Zhao et al. 2014).

For mice studies, two tailed T-tests were performed to compare results between WT and *Tnfrsf25* ^−/−^ data using Prism10. *p* < 0.05 is demoted as significant.

## Results

### Association of *TNFRSF25* CpG sites with hearing phenotypes

**Fig. 1:**
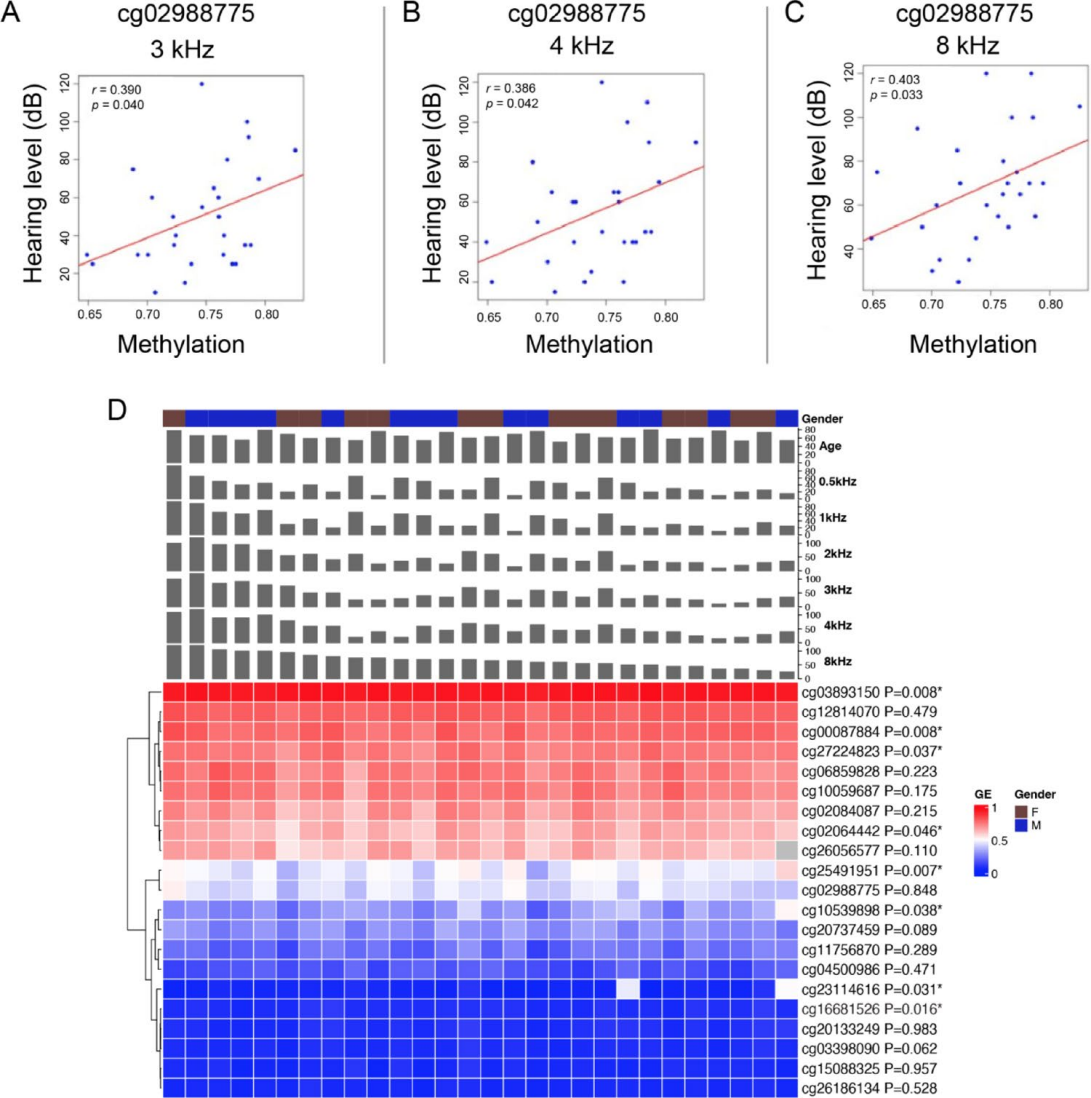
Methylation of CpGs in *TNFRSF25* is positively correlated with ARHL. **A**-**C** Positive correlation between the methylation of the CpG site (cg0298875) related to the *TNFRSF25* gene and audiometric test in ARHL patients. **D** Heatmap of methylation level of 21 CpGs of *TNFRSF25* with variables including gender, age, and audiometric test at all frequencies

Due to the technical difficulties in collecting DNA specimens from the cochlea without causing damages in hearing, the DNA isolated from the patient’ blood sample served as an alternative source for the DNA methylation analysis to determine the correlation between ARHL and DNA methylation level in patients. Linear regression analysis was used to determine the relationship between the patients hearing threshold (outcome/dependent/variable) and the m-values scores (predictor/independent variable). We observed a positive association between ARHL patients hearing thresholds and the m-values for one CpG site of The Tumor Necrosis Factor Receptor gene *TNFRSF25* (cg02988775). Specifically, the CpG site, cg02988775, (*TNFRSF25*; RefSeq: NM_003790.3) is located at the genomic positions 6,464,581–6,464,583 on the chromosome 1. There was a positive correlation with the hearing thresholds at higher audiometric frequencies (3–8 kHz) in both females and males (Fig. 1A-C). Our results indicated that the higher methylation level on this CpG site, cg02988775, was significantly correlated with the increase in the hearing threshold (Fig. 1A-C). Previously, another CpG site (cg27224823) associated with the gene *TNFRSF25* was reported to be associated with ARHL (Roche et al. 2025). We therefore analyzed the methylation level of 21 CpG sites related to *TNFRSF25* and their correlation to hearing loss. A heatmap displayed the methylation level of 21 CpG sites with other variables, including gender, age, and audiometric test at all frequencies (Fig. 1D). Eight out of 21 CpG sites were considered significantly associated with hearing loss (* *p* < 0.05; Fig. 1D). Our results suggested that the expression of the gene, *TNFRSF25*, is likely to be important to ARHL based on the correlation between the methylation level on the CpG site in this gene and the hearing loss level in ARHL patients.

### *Tnfrsf25* is expressed in mouse cochlea

**Fig. 2:**
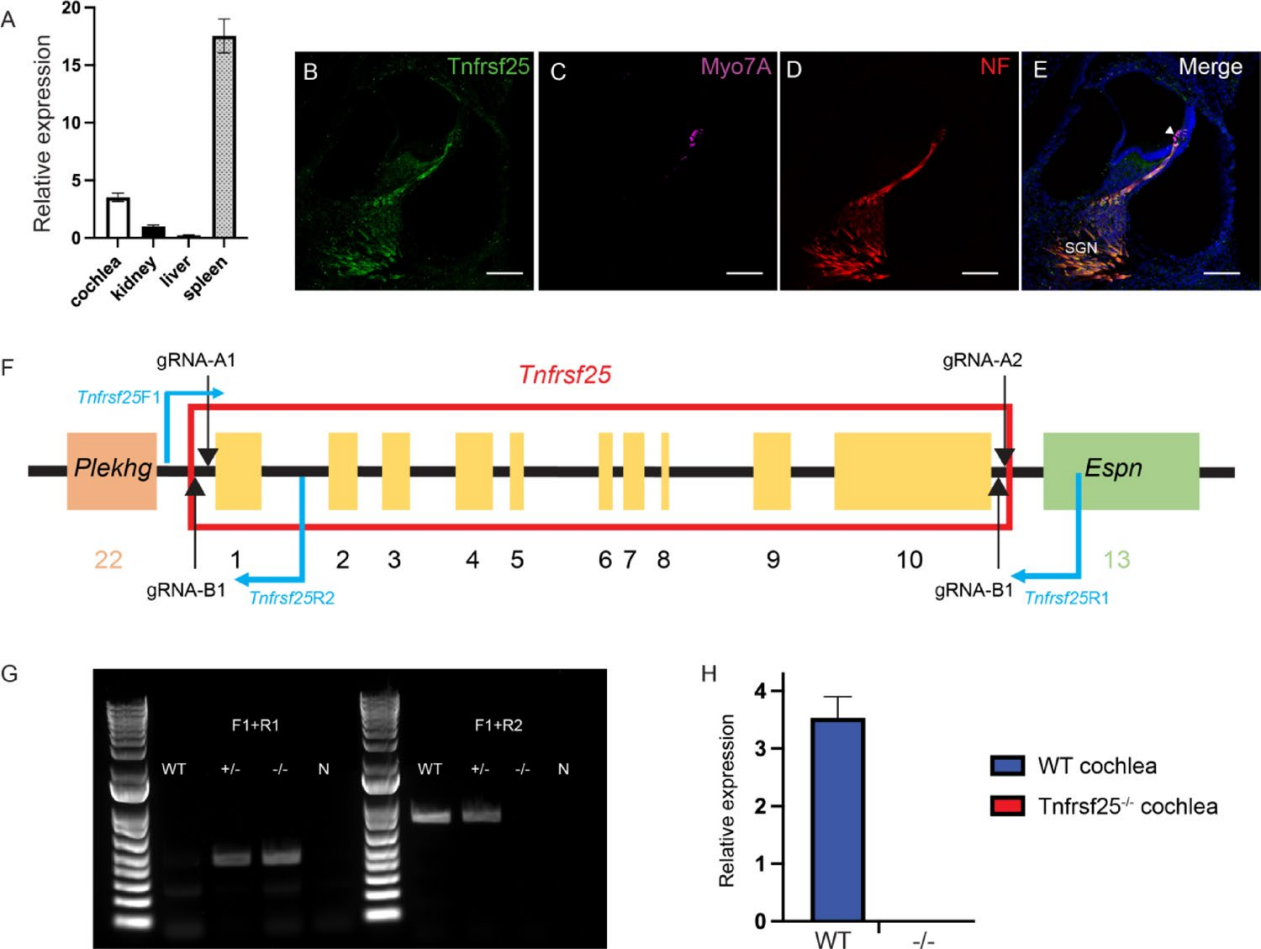
Molecular characterization. **A** qRT-PCR data of *Tnfrsf25* in various tissues of WT mouse. **B**-**E** Representative immunohistochemistry images of the cochlea in a P0 mouse. White arrowheads indicated hair cells. SGN: spiral ganglion. Scale bar = 30 μm. **F** Schematics for the designs of CRISPR/Cas9 approach and genotyping strategy. **G** Representative electrophoresis image of genotyping results. **H** Level of gene expression of *Tnfrsf25* in WT and *Tnfrsf25*^−/−^ cochlear tissues

To confirm expression of the *Tnfrsf25* in the inner ear, qRT-PCR and immunofluorescence were performed in WT mice (Fig. 2A-E). Expression data from RNAseq analysis of the gEAR database, showed the expression of *Tnfrsf25* in the murine cochlea ((Cai et al. 2015), Figure S1). To confirm this, we checked the expression of *Tnfrsf25* by qRT-PCR, and with immuno-staining of the cochlea in WT animals. qRT-PCR on RNA samples collected from 18 month-old animals showed the presence of the specific amplification product in cochlea and other organs (Fig. 2A), while confocal images of cochlear sections immunostained with anti-Myo7a, anti -Neurofilament (NFH), DAPI and anti-Tnfrsf25 receptor showed a colocalization of NFH and Tnfrsf25 in the spiral ganglion neurons (SGNs) in P0 WT Organ of Corti (Fig. 2B-D**)**. These results confirmed the expression of *Tnfrsf25* in the inner ear, supporting the possible relationship with the hearing phenotype observed in patients.

The methylation in the CpG sites commonly results in the repression or impairment in the gene expression(Stefansson et al. 2024), we hypothesized that the increased methylation in CpG sites of *TNFRSF25* leads to the ARHL by repressing the *TNFRSF25* expression. To further understand the connection between *Tnfrsf25* and hearing loss we proceeded to generate a *Tnfrsf25* knockout (*Tnfrsf25*^−/−^) mouse model. CRISPR-Cas9-mediated genome engineering was utilized to knockout the gene via deleting the exon 1 to 10 in a C57BL/6J genetic background (Fig. 2F). The genomic deletion was confirmed by PCR utilizing designed primers flanking the deleted region. The expected 446 fragment was detected in *Tnfrsf25*^*−/−*^ and Het mice, clearly showing the presence of the deleted allele, while a 742 fragment was amplified from the WT allele (Fig. 2G). These same primers were utilized for genotyping. Considering the *Tnfrsf25* gene is adjacent to the hearing loss-related gene, *Espin*, we further examined the expression of *Espin* gene to ensure the deletion of *Tnfrsf25* does not affect the expression of *Espin*. There was no statistical significance in the expression of *Espin* between WT and *Tnfrsf25*^*−/−*^ animals in 2 month- and 6 month-old age (Figure S2).

Mutant mice were viable and fertile, with the number of mice obtained for the three possible genotypes: WT, heterozygous mutant (+/-), and *Tnfrsf25*^*−/−*^ following the expected Mendelian ration (*p* > 0.05) suggesting no developmental defects nor impairments of early development. In addition, no imbalance or circling behavior were observed. Expression of *Tnfrsf25* gene was examined using qRT-PCR. Results confirmed the absence of messenger in *Tnfrsf25*^*−/−*^ mice, while gene expression was clearly observed in WT animals in the age of 18 month-old (Fig. 2H).

### *Tnfrsf25*^−/−^ mouse shows ARHL and age related loss of hair cell

**Fig. 3:**
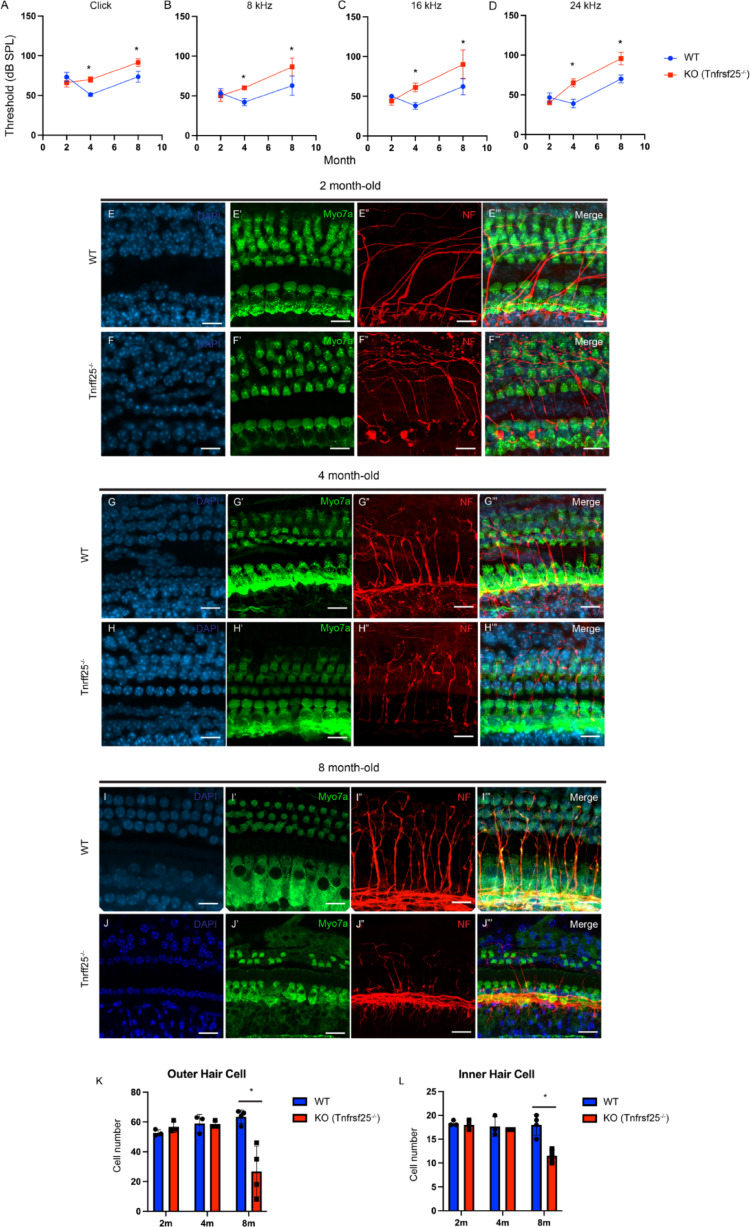
Phenotypic characterization. **A**-**D** *Tnfrsf25*^*−/−*^ mice show ARHL. Significantly increase in the ABR thresholds in *Tnfrsf25*^−/−^ mice overtime. **E**-J"' Progressive Outer and Inner Hair Cell Loss in *Tnfrsf25*^−/−^-derived cochlea. Immunohistochemistry images of outer hair cells and inner hair cells in 2 month-, 4 month- and 8 month-old WT and *Tnfrsf25*^−/−^ mice. Scale bar = 20 μm. K-L . Quantification of the cell number of outer hair cells and inner hair cells in the cochlea of 2 month-, 4 month- and 8 month-old WT and *Tnfrsf25*^−/−^ mice (*N* ≥ 3). *: *p* < 0.05

To assess whether *Tnfrsf25* deletion impacted hearing, auditory brainstem response (ABRs) tests were performed in WT and *Tnfrsf25*^*−/−*^ mice (N≥ 3 per genotype; Figures S3-S5). There was no difference in the ABR thresholds on any tested frequency between WT and *Tnfrsf25*^*−/−*^ mice at the age of two months (Fig. 3A-D). At 4 months of age, the ABR thresholds for click response were significantly different for both genotypes. While in WT was 51 ± 2.24 dB SPL (decibel sound pressure level), in *Tnfrsf25*^*−/−*^ mice was 70 ± 3.54 dB (*p* < 0.05) (Fig. 3A). Likewise, for the pure tone frequencies, *Tnfrsf25*^*−/−*^ mice showed significantly elevated ABR thresholds compared to WT mice (*p* < 0.05). There was 42 ± 4.48 dB vs. 60 ± 0 dB, 38 ± 4.47 dB vs. 61 ± 5.48 dB, and 39 ± 5.47 vs. 65 ± 5 at 8, 16, and 24 Hz, respectively (Fig. 3B-D). Progressively at 8 months of age, the ABR threshold for thresholds for click response in WT was 74 ± 6.90 dB SPL, which was significantly lower than the threshold in *Tnfrsf25*^*−/−*^ mice to 91 ± 4.76 dB as well (Fig. 3A). The pure tone thresholds across three different frequencies, 8 (63 ± 12.20 vs. 84 ± 11.07; *p* < 0.05), 16 (62 ± 10.35 vs. 90 ± 18.26, *p* < 0.05), and 24 Hz (70 ± 5.00 vs. 96 ± 7.87; *p* < 0.05), in *Tnfrsf25*^*−/−*^ mice were significantly higher compared to the thresholds in WT mice (*n* = 7; Fig. 3B-D). Moreover, we observed a significantly increase in the ABR thresholds between 4 month-old animals and 8 months-old animals (*p* < 0.05) suggesting that the ARHL was exhibited the *Tnfrsf25*^*−/−*^ mice (Fig. 3A-D). Though the amplitude and latency time for the wave I of the ABR did not show the significant different, we nevertheless observed the trends of larger amplitude in WT and longer latency time in *Tnfrsf25*^*−/−*^, especially in 8 month-old animals (Figure S6-7).

To study the pathophysiology of the ARHL related to the lack of *Tnfrsf25*, we performed immunohistochemistry using anti-Myo7a and anti-NF antibody in the middle turn of cochlea. There were no noticeable hair cells differences between the *Tnfrsf25*^*−/−*^ and WT at 2 month- and 4 month-old age (Fig. 3E-H**”’**). However, we observed the less nerve innervation with obviously crooked nerve phenotype in the 4 month-old *Tnfrsf25*^*−/−*^ cochlea compared to WT animals in the same age (Fig. 3G**” **& J”; N = 5). At 8 month-old, we observed the signs of degeneration in nerve invasion as well along with the loss of outer hair cells (OHCs) in *Tnfrsf25*^*−/−*^ mouse (Fig. 3I-J**’”**). Statistically, there was no difference in the numbers of OHC and IHC between WT and *Tnfrsf25*^*−/−*^ cochlea at the age of 2 and 4 month-old (Fig. 3K-L). However, significance differences were seen in OHC (WT vs *Tnfrsf25*^*−/−*^ = 63.5 ± 4.5 vs 26.8 ± 17; *p* < 0.05) and IHC (18 ± 2.2 vs 11.5 ± 1.3; *p* < 0.05) across ~ 130 microns in 8 month-old cochlea (Fig. 3K-L). The loss of hair cells and the decreased nerve innervation may contribute to the ARHL observed in ABR tests.

## Discussion

TNFRSF25 (TNF Receptor superfamily Number 25) protein is a member of the tumor necrosis factor receptor (TNFR) family, also known as DR3. TNFRSF25 is a cell-surface protein that interact with a corresponding TNF-related ligand family via extracellular cysteine-rich domain (Valatas et al. 2019). This gene has been found to play a significant role in apoptosis and the death signaling pathway (Schreiber et al. 2011). Hearing function is dependent on fluid and ion homeostasis in the inner ear, and these processes are maintained by cochlear microcirculation. It has been shown that disturbance in cochlear microcirculation caused by inflammation, via the release of the inflammatory cytokine tumor necrosis factor alpha (TNF-alpha) can cause inner ear disorders such as late onset hearing loss, presbycusis and tinnitus (Ihler et al. 2013). In addition, high levels of TNF-alpha can potentially cause damage to hair cells within the cochlear by apoptosis and disrupt cellular homeostasis (Wu et al. 2022). TNF-alpha has a regulatory role in the cochlea, can be induced with ageing (Riva et al. 2007) and, in response to noise-exposure, this gene may initiate inflammatory responses in the cochlear structure playing a role in the mechanism of noise-induce cochlear damage (Fujioka et al. 2006). This evidence suggests that the pro-inflammatory cytokine plays an important role in the inner ear function.

Transcription factors are drivers of gene regulation, and epigenetic modifications regulates their ability to target genes (Layman and Zuo 2014). The inner ear of mammals consists of the cochlea, a key player in the sense of hearing, the vestibule and three semicircular canals involved in the sense of balance. DNA methylation patterns in the cochlea undergo many changes during development and have been involved in regulating the differentiation and maturation processes of cochlear hair cells and supporting cells (Nguyen et al. 2023). DNA methylation involves the addition of a methyl group to the fifth residues in CpG dinucleotides leading to transcriptional repression. CpG site methylation also plays a role in the development and function of the inner ear within the cochlea sensory epithelium (Mittal et al. 2020).

Our data from a cohort of 30 ARHL patients showed an association between elevated CpG methylation level in a CpG sites located in the *TNFRSF25* gene with audiometric hearing thresholds of presbycusis patients, suggesting that this site-specific change in methylation may affect *TNFRSF25* expression levels. We hypothesized that increase methylation level on the CpG site (cg02988775) may reduce the expression of *TNFRSF25* as methylation in CpG sites are commonly associated with the repression in the gene expression (Stefansson et al. 2024). We proceeded with generating a mouse model to further study the role of *Tnfrsf25* in hearing. We found that *Tnfrsf25* is expressed in the cochlea, co-localizing with the NF, a marker for neuronal cells, suggesting a potential role in the mechanics of sound perception. The generation of *Tnfrsf25*^*−/−*^ mice revealed that animals lacking the *Tnfrsf25* develop ARHL overtime. The abnormal nerve innervation pattern was first observed in the 4 month-old *Tnfrsf25*^*−/−*^ specimens without the loss of hair cells. At 8 months of age *Tnfrsf25*^*−/−*^ mice revealed a significant loss of hair cells along with the reduced nerve innervation, when compared with WT mice. Based on the immunostaining showing the colocalization of Tnfrsf25 and NF, especially in the spiral ganglion, we hypothesized that the loss of hair cells is the consequence of primary degeneration of nerve and the reduced nerve innervation. Further study is necessary to determine the mechanism underlying the degeneration driven by the lack of *Tnfrsf25*. Nevertheless, these experiments have proven the progressive hearing loss and cochlear impairments in the *Tnfrsf25*^*−/−*^ mouse model, which suggested the role of *Tnfrsf25* gene in the maintenance of the cochlear function.

We further noticed that the cellular phenotypes of *Tnfrsf25*^*−/−*^ cochlear are strikingly similar to animal with the conditional knockout of the *Brd4* gene in hair cells (Kannan-Sundhari et al. 2020). The Brd4 is a member of the bromodomain and extraterminal (BET) family of proteins. Brd4 protein is known to regulate gene expression epigenetically by binding to acetylated histone and recruit other proteins (Filippakopoulos and Knapp 2014, Wernersson et al. 2022). Mutations in the *Brd4* gene result in various diseases including cancers and neurological disorders (Hamad et al. 2022, Hu et al. 2022, Jouret et al. 2022). In the inner ear, the lack of *Brd4* in cochlear hair cells leads to the progressive degeneration of hair cells from P8 followed by the reduction in the nerve innervation (Kannan-Sundhari et al. 2020), which supported that the epigenetic regulation is important in the survival of cochlear cells and maintenance of normal auditory function by directly altering the epigenetic regulator. On the other hand, this current study demonstrated the effect of methylation level in genes on the ARHL. Multiple studies highlighted that, even though it may undergo different mechanisms, epigenetic regulation is the critical player in ARHL. Overall, we have established a potential role for *TNFRSF25* in cochlear function and maintenance related to ARHL, with a mechanism that involves epigenetic regulation of the target gene.

## Supplementary Information

Below is the link to the electronic supplementary material.

Supplementary Material 1

## Data Availability

The datasets generated and/or analyzed during the present study are available from the corresponding author on reasonable requests.
