# Supplementary material for: The involvement of TNFRSF25 in age-related hearing loss
Source: Hum Genet. 2026 Mar 26;145(1):33. doi: 10.1007/s00439-026-02826-5 (PMC13018069; doi:10.1007/s00439-026-02826-5)
Supplement: Supplementary file 1 — Supplementary Material 1 [file 439_2026_2826_MOESM1_ESM.docx]

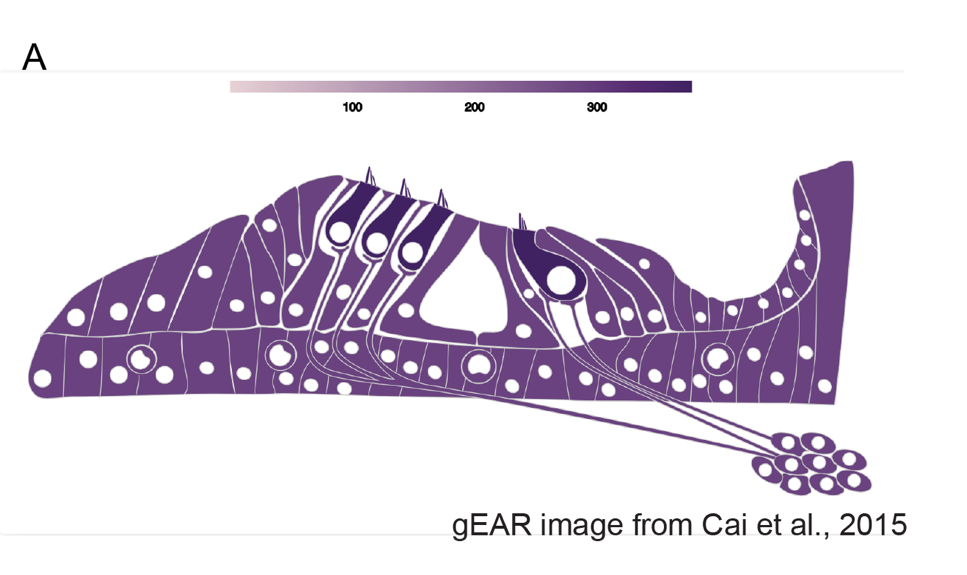


Figure S1. Gene expression data of *Tnfrsf25* from gEAR database (PMID: 25855195) based on RNAseq data in P0 mouse cochleae (Cai et al., 2015).


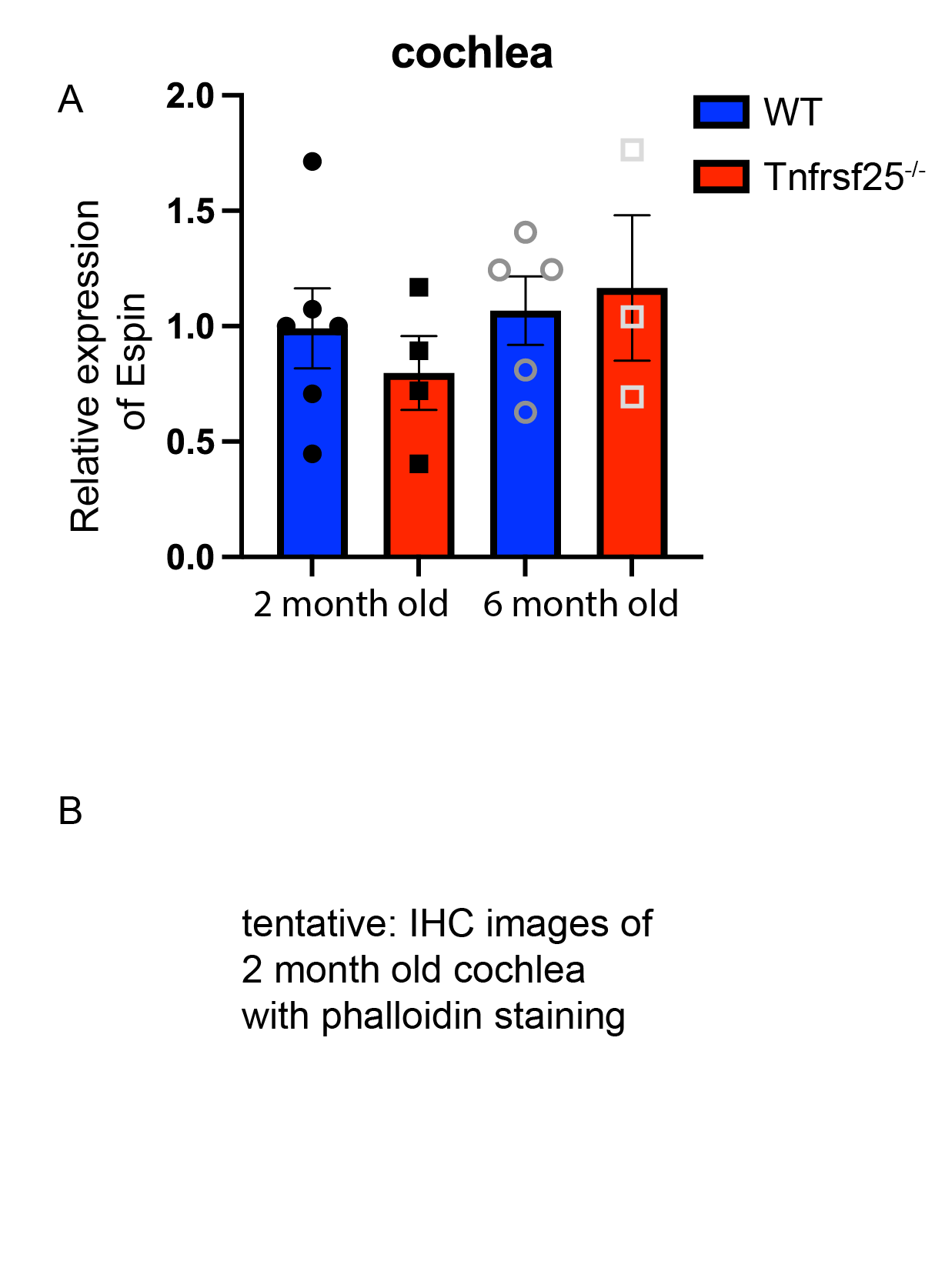


Figure S2. qRT-PCR showed the comparable expression of *Espin* between WT and Tnfrsf25^-/-^ animals at 2 month- and 6-month old age (*p* < 0.05).


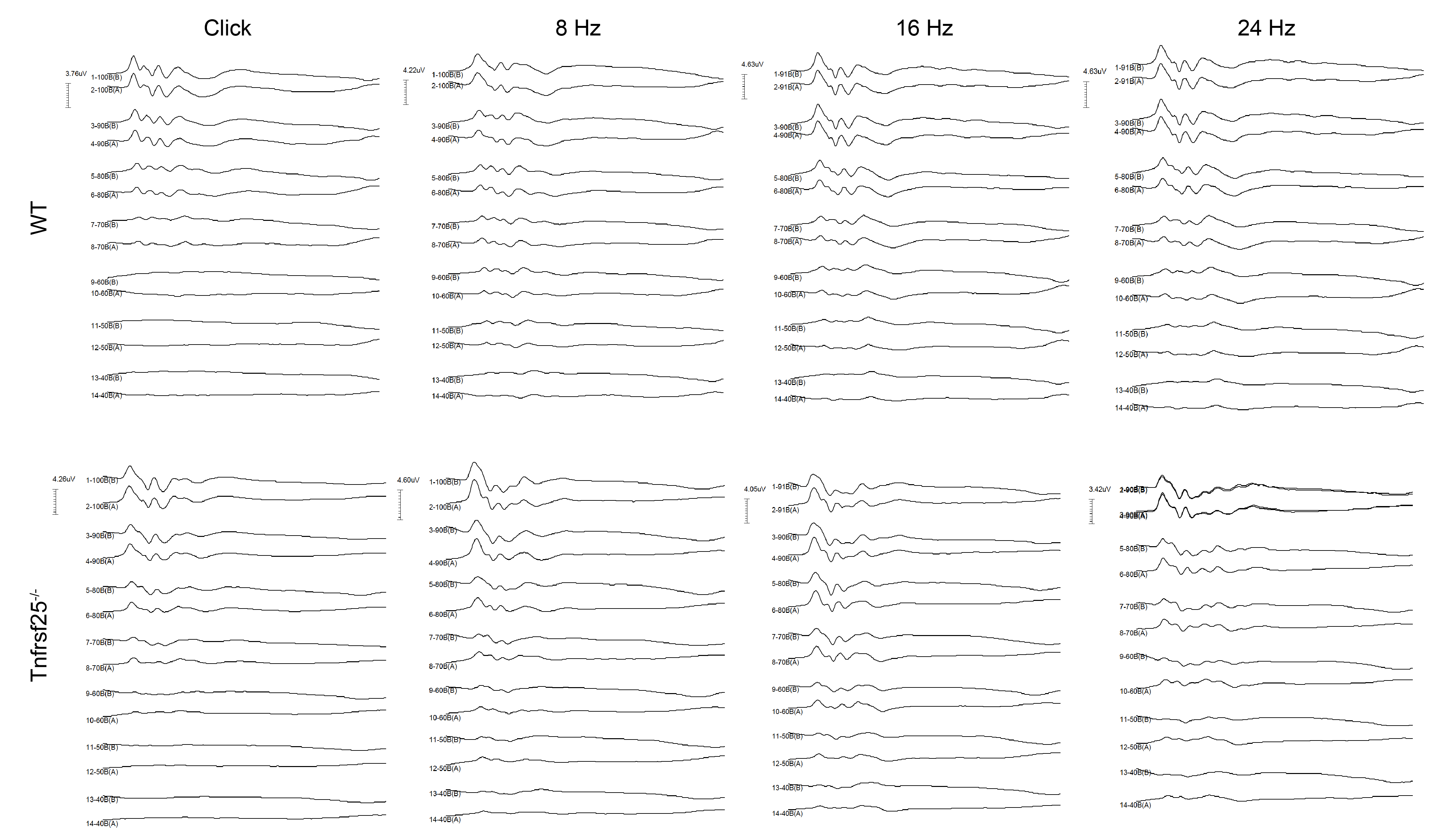


Figure S3. Representative ABR waveforms from 2 month-old animals


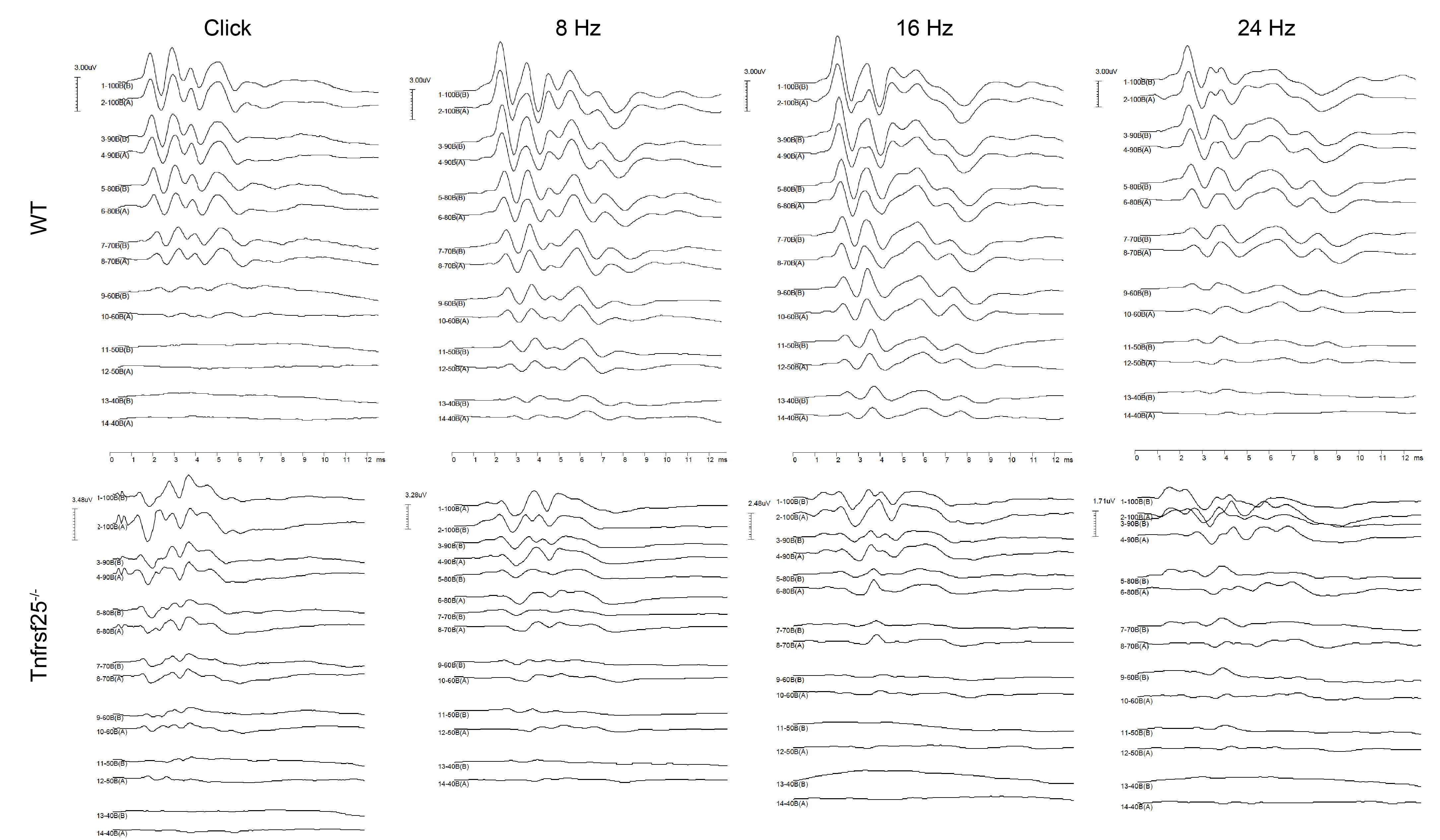


Figure S4. Representative ABR waveforms from 4 month-old animals


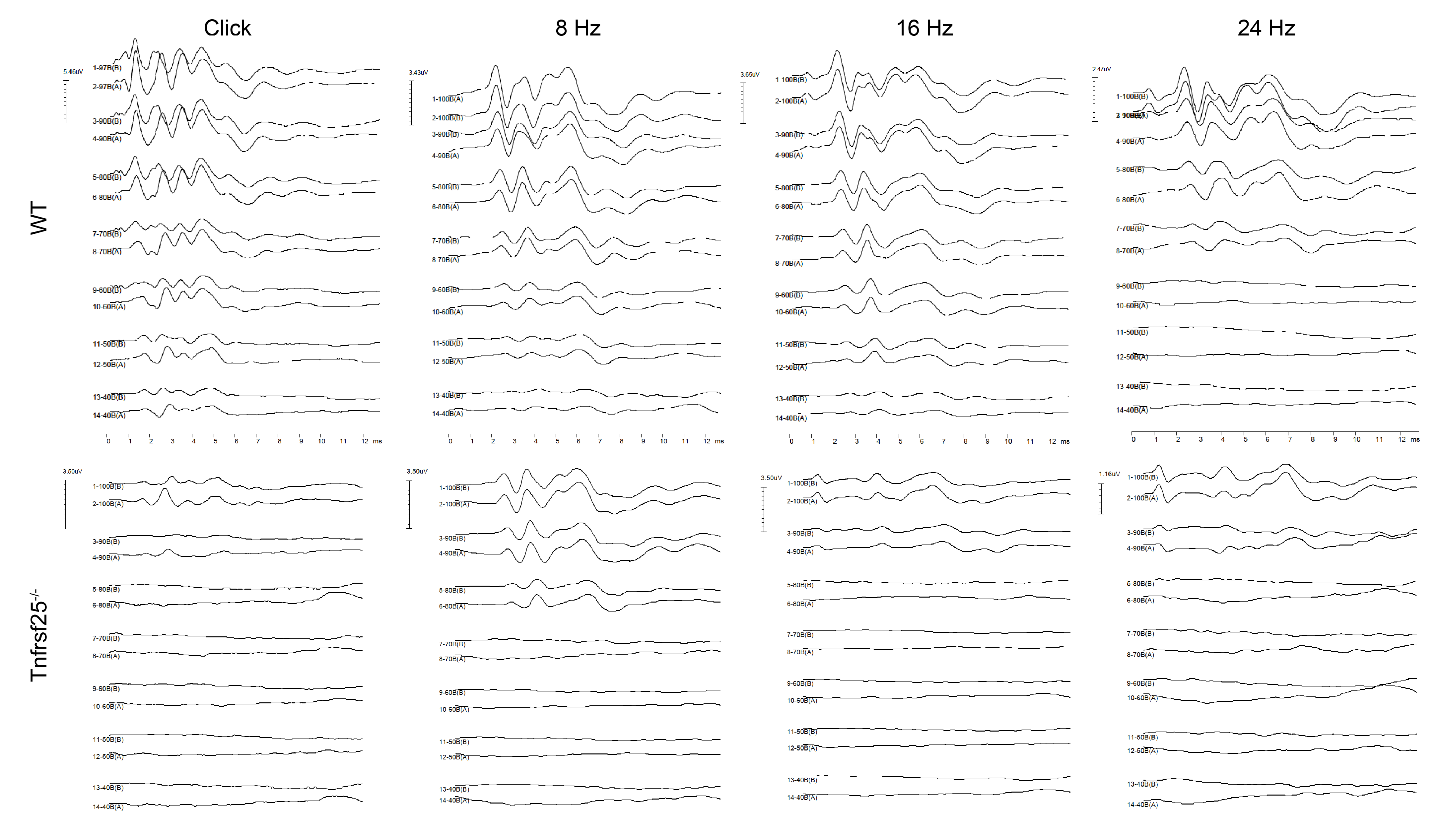


Figure S5. Representative ABR waveforms from 8 month-old animals


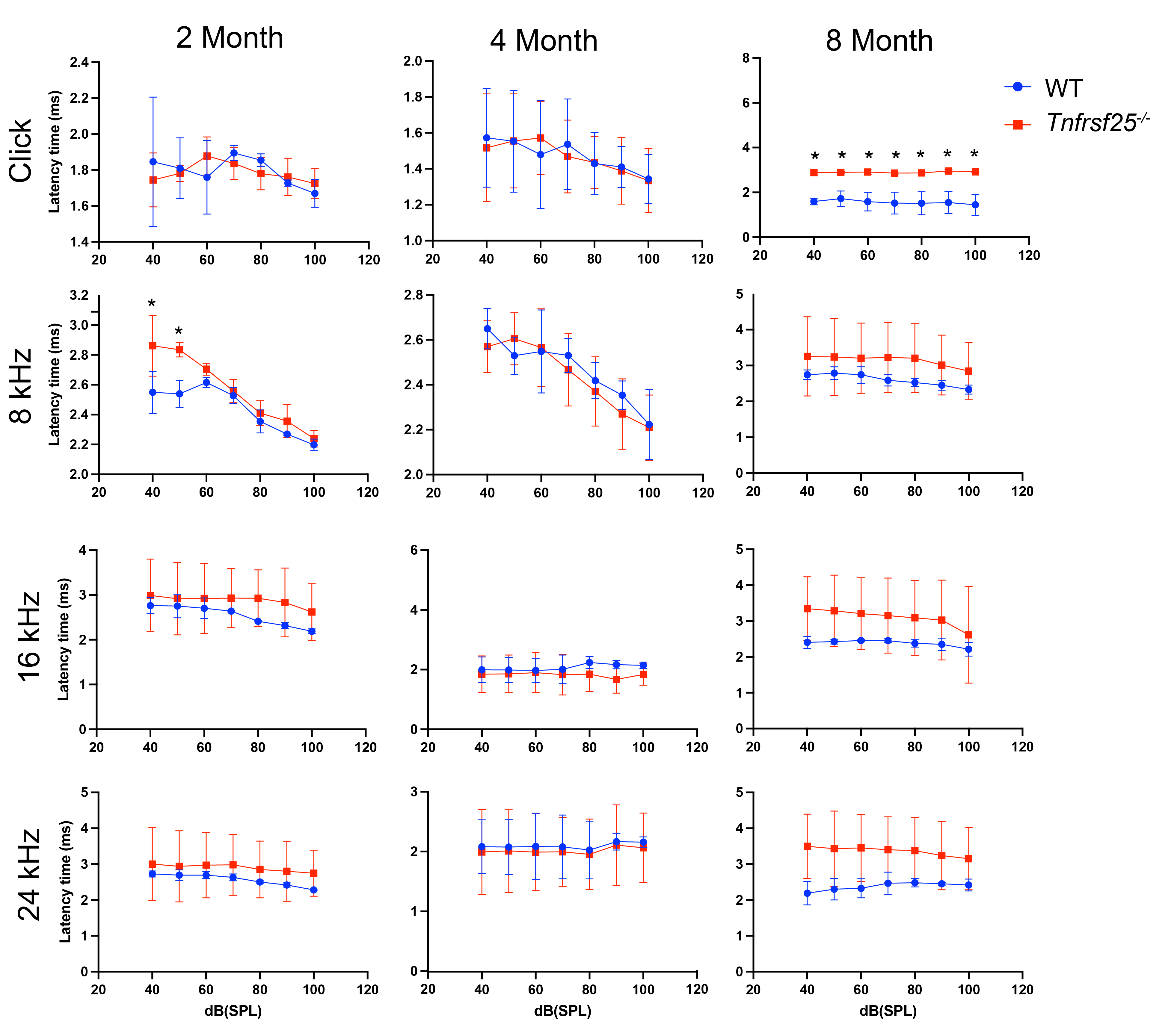


Figure S6. Latency time for the wave I. * *p*<0.05. Error bars are standard deviations.


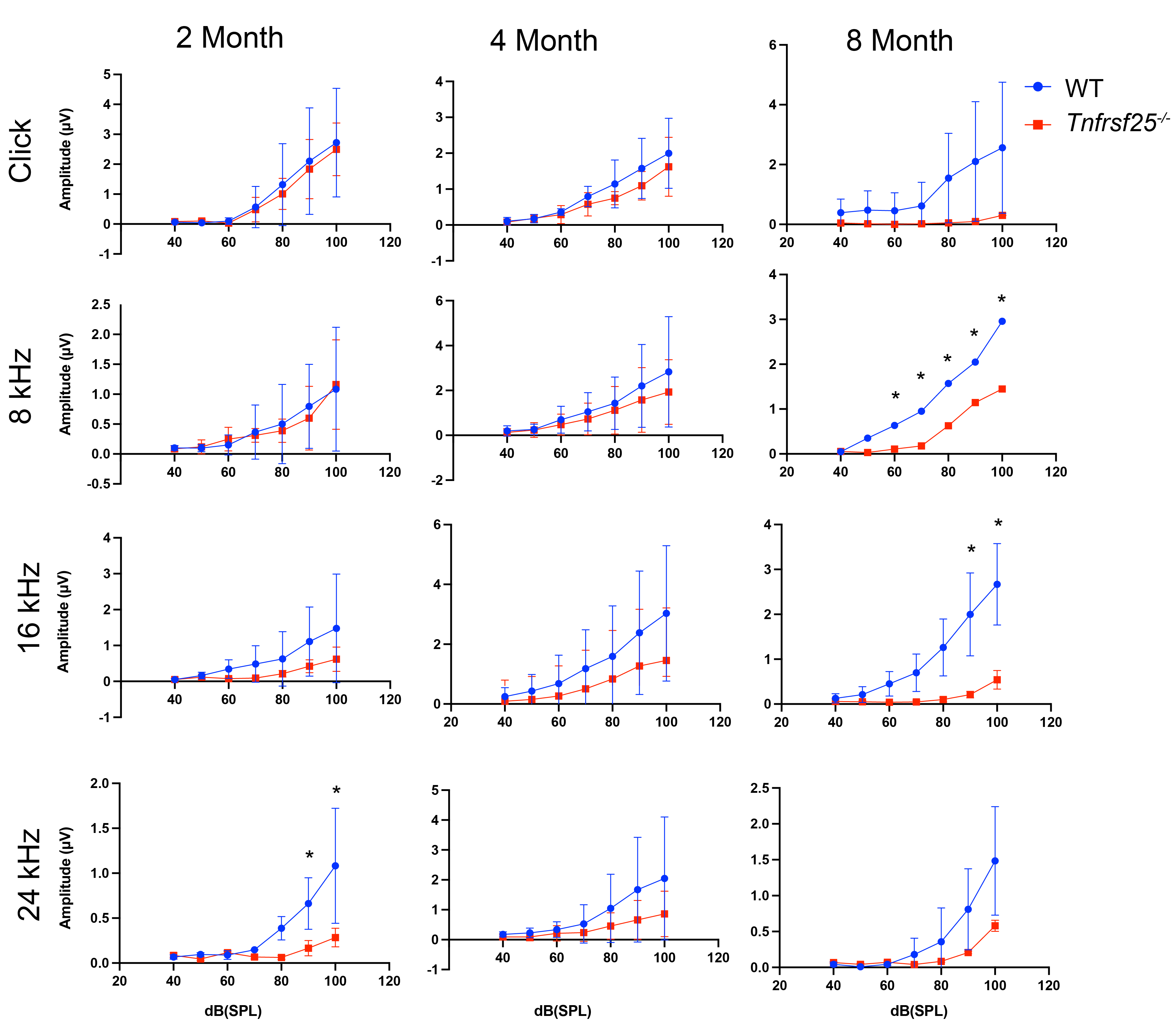


Figure S7. Amplitude for the wave I. * *p*<0.05. Error bars are standard deviations.

Table S1. gRNA sequences for the generation of *Tnfrsf25* Knock-out mouse.

| Name | gRNA sequence | PAM sequence |
| --- | --- | --- |
| gRNA-A1 | CTATGTCCCTCTACGTGACG | GGG |
| gRNA-A2 | TCCTGACGCCTCGGGAACTG | AGG |
| gRNA-B1 | GTTGCACCCTGATCCCACGC | TGG |
| gRNA-B2 | AGTGGGAGTTAAGGGCTCTC | TGG |
